# No benefit from TMZ treatment in glioblastoma with truly unmethylated *MGMT* promoter: Reanalysis of the CE.6 and the pooled Nordic/NOA-08 trials in elderly glioblastoma patients

**DOI:** 10.1093/neuonc/noae108

**Published:** 2024-07-03

**Authors:** Monika E Hegi, Felix B Oppong, James R Perry, Wolfgang Wick, Roger Henriksson, Norman J Laperriere, Thierry Gorlia, Annika Malmström, Michael Weller

**Affiliations:** Neurosurgery & Neuroscience Research Center, Lausanne University Hospital and University of Lausanne, Lausanne, Switzerland; European Organisation for Research and Treatment of Cancer (EORTC) Headquarters, Brussels, Belgium; Odette Cancer Centre, Sunnybrook Health Sciences Centre, Toronto, Ontario, Canada; DKTK and Clinical Cooperation Unit Neurooncology, DKFZ, Heidelberg, Germany; Neurology Clinic and National Center for Tumor Diseases, University Hospital Heidelberg, Heidelberg, Germany; Department of Radiation Sciences, Oncology, Umeå University, Umeå, Sweden; Radiation Oncology, Princess Margaret Cancer Centre, Toronto, Ontario, Canada; European Organisation for Research and Treatment of Cancer (EORTC) Headquarters, Brussels, Belgium; Department of Advanced Home Care in Linköping, and Division of Cellbiology, Department of Biomedical and Clinical Sciences, Linköping University, Linköping, Sweden; Department of Neurology & Brain Tumor Center, University Hospital and University of Zurich, Zurich, Switzerland

**Keywords:** elderly/frail GB patients, *MGMT* promoter methylation, stratified treatment

## Abstract

**Background:**

The treatment of elderly/ frail patients with glioblastoma is a balance between avoiding undue toxicity, while not withholding effective treatment. It remains debated, whether these patients should receive combined chemo-radiotherapy with temozolomide (RT/TMZ→TMZ) regardless of the O^6^-methylguanine DNA methyltransferase gene promoter (*MGMTp*) methylation status. MGMT is a well-known resistance factor blunting the treatment effect of TMZ, by repairing the most genotoxic lesion. Epigenetic silencing of the *MGMTp* sensitizes glioblastoma to TMZ. For risk-adapted treatment, it is of utmost importance to accurately identify patients, who will not benefit from TMZ treatment.

**Methods:**

Here, we present a reanalysis of the clinical trials CE.6 and the pooled NOA-08 and Nordic trials in elderly glioblastoma patients that compared RT to RT/TMZ→TMZ, or RT to TMZ, respectively. For 687 patients with available *MGMT*p methylation data, we applied a cutoff discerning truly unmethylated glioblastoma, established in a pooled analysis of 4 clinical trials for glioblastoma, with RT/TMZ→TMZ treatment, using the same quantitative methylation-specific *MGMTp* PCR assay.

**Results:**

When applying this restricted cutoff to the elderly patient population, we confirmed that glioblastoma with truly unmethylated *MGMTp* derived no benefit from TMZ treatment. In the Nordic/NOA-08 trials, RT was better than TMZ, suggesting little or no benefit from TMZ.

**Conclusions:**

For evidence-based treatment of glioblastoma patients validated *MGMTp* methylation assays should be used that accurately identify truly unmethylated patients. Respective stratified management of patients will reduce toxicity without compromising outcomes and allow testing of more promising treatment options.

For the podcast associated with this article, please visit ‘https://soc-neuro-onc.libsyn.com/tmz-and-mgmt-in-elderly-gbm-patients’

**Key Points**

- No benefit from TMZ in GB with truly unmethylated *MGMTp.*
- Improved management of elderly/frail GB patients to avoid undue toxicity without compromising outcomes.
- Patient selection for clinical trials omitting TMZ.

**Importance of the Study**

*MGMTp* methylation testing is controversial, which limits stratified therapy to reduce unwanted toxicity or select patients into clinical trials omitting temozolomide. Here we reanalyzed *MGMTp* methylation data of 3 phase III trials treating elderly/frail patients with RT versus TMZ or RT versus RT/TMZ→TMZ. The pooled analysis of quantitative *MGMT* methylation-specific PCR data from these patients allowed validation of an unsupervised cutoff and a lower supervised cutoff, informed by outcome (cutoff with safety margin) previously determined in 4 GB trials using the same assay. The cutoff with the safety margin defines a “gray zone” comprising patients with low *MGMTp* methylation, who performed significantly better than truly unmethylated patients. Validation of this cutoff with a safety margin for elderly/frail patients is suitable for risk-adjusted patient management.

Treatment of glioblastoma patients is a challenge and the core of the current standard of care remains radiotherapy (RT) and temozolomide (TMZ) treatment, for almost 20 years. O^6^-methylguanine DNA methyltransferase (MGMT) is a well-known resistance factor that blunts the treatment effect of TMZ, by repairing the most toxic lesion.^1,2^ In almost 50% of patients with glioblastoma (GB) the *MGMT* gene promoter (*MGMTp*) is epigenetically silenced by methylation, while the second *MGMT* copy is usually lost in GB due to the recurrent loss of one copy of chromosome 10. *MGMTp* methylation of the tumor has been shown to be predictive for benefit from TMZ in GB patients^3–5^ and is therefore stratified for clinical trials. Moreover, an unmethylated *MGMTp* status is used as a biomarker to select patients into clinical trials that omit TMZ treatment to avoid undue toxicity, when testing new drugs.^6–8^ This has raised the question of the correct cutoff, in order not to withhold TMZ from patients who potentially could benefit from it. Some trials have used the technically motivated cutoff set at the nadir of the bimodal distribution.^9^ Since the uncertainty at the nadir is high, other studies have opted for a safety margin to select only “truly” unmethylated patients, choosing the lower bound of the 95% confidence interval.^10,11^ The relevance of the lower bound cutoff for predicting benefit from TMZ was substantiated statistically in a pooled analysis including over 2000 GB patients treated in 4 clinical trials with RT/TMZ→TMZ.^12^ This newly defined safety margin, determined with overall survival (OS) supervised analysis in this large cohort, corresponded to a 96% chance of being *MGMTp* unmethylated, hence, allowing the identification of “truly” *MGMTp* unmethylated patients.

In the standard of care practice, most GB patients receive combination therapy with TMZ, regardless of the *MGMTp* methylation status. However, in elderly and frail patients it may be considered to treat with either TMZ or RT only, which has been tested in 2 phase III trials. The *MGMTp* methylation analysis was supportive of the predictive value for benefit from TMZ treatment, indicating that unmethylated patients likely did not benefit from TMZ treatment, and it was proposed to treat with RT instead.^4,5^ A third study compared a combination of a short course RT with or without concomitant and adjuvant TMZ (RT/TMZ→TMZ).^13^ The *MGMTp* methylation analysis confirmed the predictive value. However, the combination therapy in the unmethylated patients showed an apparent better outcome that almost reached statistical significance when compared to the RT-arm (*P* = .055). This raised the question of whether all patients should get the combination treatment with TMZ regardless of the *MGMTp* methylation status, in order not to undertreat patients.

In order to clarify this question and to protect frail patients from undue toxicity, we obtained the raw data of the quantitative methylation-specific PCR (qMSP) assays that were performed centrally for each of the 3 aforementioned trials treating elderly patients. We reanalyzed the *MGMTp* methylation data by applying the cutoff with the safety margin.^12^ Here we report that “truly” *MGMTp* unmethylated patients do not benefit from TMZ, neither administered alone, nor when added to RT.

## Materials and Methods

### Data Selection

Quantitative *MGMTp* methylation data were obtained from 3 clinical trials treating elderly patients with newly diagnosed GB. All 3 trials used the same qMSP assay performed centrally.^14^ qMSP raw data with valid results were available from a total of 687 patients out of 1276 patients. Patients were randomized in the Nordic trial^4^ to TMZ (200 mg/m^2^ days 1–5 every 4 weeks for a maximum 6 cycles) or 1 of the 2 doses of RT (60 Gy, in 30 fractions of 1.8–2.0 Gy, or hypofractionated, 3.4 Gy fractions to 34 Gy; Trials registration number, ISRCTN81470623). In the NOA-08 trial^5^ patients were randomized to TMZ (TMZ 100 mg/m^2^ 7 days on-7 days off) or RT (60 Gy, in 30 fractions of 1.8–2.0 Gy; Clinical Trial.gov NCT01502241). In the CE.6 trial^13^ (CCTG CE.6, EORTC 26062-22061, TROG03.01) patients were randomized to short-course RT (40 Gy in 15 fractions over 3 weeks) with or without concomitant and adjuvant TMZ (RT/TMZ→TMZ, 75 mg/m^2^/day for 21 days; 150–200 mg/m^2^, 5 days/28-day cycle, for up to 12 cycles or progression; NCT00482677). For this study, the data from the Nordic and NOA-08 trials were pooled (*n* = 329), and stratified by treatment (TMZ, *n* = 133; RT, 196) regardless of differences in treatment schedule and dose. For the CE.6 trial data were available for 359 patents (RT/TMZ→TMZ, *n* = 183; RT, *n* = 175).

### Ethics Approval and Consent to Participate

Patients from the clinical trials cohorts, provided written informed consent for translational research in the context of their enrollment into the clinical trial and the study was approved by the ethics committees of the participating centers.^4,5,13^ This study has been performed under institutional and international guidelines and regulations as previously reported. The study was conducted in accordance with the Declaration of Helsinki.

### qMSP Assay and Analysis

The qMSP assay was performed and analyzed centrally as described using the same qMSP assay.^4,5,13,14^ For the present analysis the raw copy number data of the qMSP for *MGMT* and *β-actin* (ACTB) was obtained and served as input to calculate the “corrected” *MGMT* methylation ratio (*Ratio*_*c*_)^12^: $\;Ratio_{C}=log_{2}\left(1000\times \frac{MGMT+1}{ACTB}\right)$. This “corrected” procedure adds one copy of *MGMT* to the numerator as compared to the original calculation^14^ to retain samples with zero methylated *MGMT* copies that otherwise would be lost upon logarithmic transformation. Classification of the samples was according to the following rules:

- ACTB ≥ 1250:
  ◦ MGMT < 10: unmethylated
  ◦ MGMT ≥ 10: status according to $Ratio_{c}$, ie, methylated if ≥ 1, otherwise unmethylated.
- ACTB < 1250:
  ◦ MGMT < 10: invalid sample
  ◦ MGMT ≥ 10: methylated

### Distribution of *MGMTp* Methylation Ratio and Classification

A bimodal Gaussian mixture model was applied to determine the distribution of the *MGMTp* methylation log2 ratios in the datasets.

### Statistical Analyses

**Figure 1.**
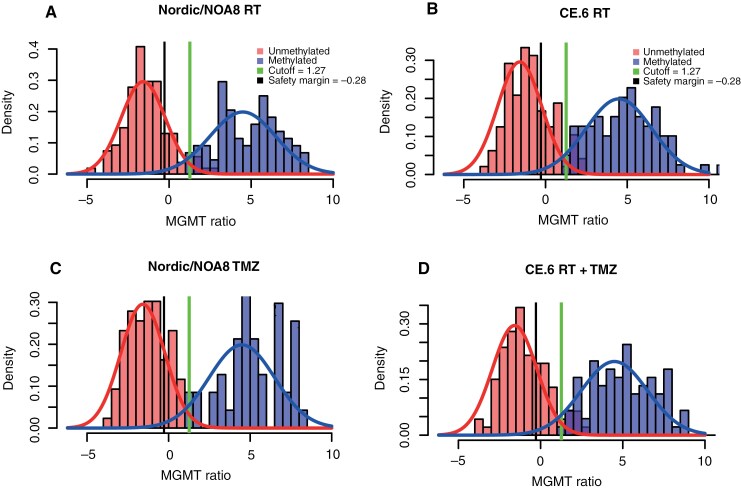
Bimodal distribution of *MGMTp* methylation in trial populations of elderly GB patients. The histograms of the corrected MGMT ratio (Log2 [1000 × (*MGMT *+ 1)/*ACTB*)]) shown by the trial/treatment arm indicate that the previously determined cutoff of 1.27 (indicated with a vertical line) is a good fit, close to the intersection of the methylated and unmethylated *MGMT* ratio. The corresponding safety margin of −0.28 is indicated with a vertical line. The overlap between the distribution of *MGMTp* unmethylated and *MGMTp* methylated is marked. The area between the cutoff (1.27) and the safety margin (−0.28) is defined as a gray zone, delineated by vertical lines.

The patients’ valid qMSP results were subjected to the “corrected” classification using the cutoff of 1.27 and a safety margin of −0.28.^12^ Using this cutoff for the “corrected” *MGMTp* methylation ratio, samples were classified as unmethylated if the ratio was less than the cutoff and methylated if otherwise. The comparability of the *MGMTp* methylation classification results between the assays was quantified using the Cohen Kappa coefficient. Values between the cutoff and the safety margin were defined as the gray zone (Figure 1). This gray zone comprises the samples in the tail of the Gaussian distribution of “methylated” that overlapped with the distribution of the unmethylated samples, and therefore cannot be classified with high certainty to be “truly” unmethylated. Subsequently, samples that were below the safety margin of −0.28 were classified as “truly” unmethylated. Overall survival analysis was performed using the Kaplan–Meier method.^15^ Progression-free survival analysis was performed in the NOA-08 and CE.6 trials, no progression data was available in the Nordic trial as this was not collected. SAS version 9.4 (© 2002-2012 per SAS Institute Inc., Cary, NC, USA) was used for the Cox models.

## Results

### Patient Characteristics

The baseline characteristics of the 687 patients with valid *MGMTp* methylation data included in this analysis are presented in Table 1. The median age was 72 years, ranging from 60 to 90 years, with over 62% of the patients older than 70 years. The majority of the patients (82%) had their tumors resected, and more than 80% of the patients had a WHO performance status of 0 or 1.

**Table 1.** Patient’s Baseline Characteristics

	Trial/treatment				Total (*N* = 687)
	Nordic/NOA8/RT (*N* = 196)	Nordic/NOA8/TMZ (*N* = 133)	CE.6/RT (*N* = 175)	CE.6/RT + TMZ (*N* = 183)	
	*N* (%)	*N* (%)	*N* (%)	*N* (%)	*N* (%)
***Sex***					
Male	102 (52.0)	85 (63.9)	97 (55.4)	110 (60.1)	394 (57.4)
Female	94 (48.0)	48 (36.1)	78 (44.6)	73 (39.9)	293 (42.6)
*Age*					
Median	70.1	71.0	74.0	73.0	72.0
Range	60.5–83.6	60.1–83.1	65.0–88.0	65.0–90.0	60.1–90.0
Q1–Q3	67.0–73.5	67.9–74.9	70.0–76.0	69.0–76.0	68.8–75.0
*Age (categorized)*					
≤70	94 (48.0)	58 (43.6)	48 (27.4)	58 (31.7)	258 (37.6)
> 70	102 (52.0)	75 (56.4)	127 (72.6)	125 (68.3)	429 (62.4)
*Type of surgery*					
Biopsy	39 (19.9)	25 (18.8)	27 (15.4)	32 (17.5)	123 (17.9)
Resection	157 (80.1)	108 (81.2)	148 (84.6)	151 (82.5)	564 (82.1)
*WHO performance status*					
0	70 (35.7)	30 (22.6)	37 (21.1)	55 (30.1)	192 (27.9)
1	90 (45.9)	75 (56.4)	108 (61.7)	86 (47.0)	359 (52.3)
2	36 (18.4)	28 (21.1)	30 (17.1)	42 (23.0)	136 (19.8)
^a^ *MGMT (qMSP)*					
Unmethylated	100 (51.0)	86 (64.7)	96 (54.9)	93 (50.8)	375 (54.6)
Methylated	95 (48.5)	47 (35.3)	77 (44.0)	88 (48.1)	307 (44.7)
^b^Invalid	1 (0.5)	0 (0.0)	2 (1.1)	2 (1.1)	5 (0.7)
*Survival status*					
Alive	10 (5.1)	19 (14.3)	7 (4.0)	11 (6.0)	47 (6.8)
Dead	186 (94.9)	114 (85.7)	168 (96.0)	172 (94.0)	640 (93.2)

^a^MGMT-status as reported with original *MGMTp* methylation classification procedure. 589 patients were reported to have missing or invalid qMSP data, 205 patients in Nordic/NOA8/RT, 180 in Nordic/NOA8/TMZ, 106 in CE.6/RT and 98 CE.6/RT + TMZ.

^b^5 patients had valid qMSP values, but were classified as invalid using the original *MGMTp* methylation classification procedure, and could be reclassified using the “corrected” *MGMTp* methylation ratio (*Ratio*_*c*_).

#### Application of cutoff of 1.27 and safety margin of −0.28.—

The density plot visualized in Figure 1 revealed that the cutoff at 1.27 was a good fit for the GB of this elderly patient population, with the cutoff close to the intersection of the methylated and unmethylated *MGMT*p ratio (nadir). The number of patients classified into *MGMTp* truly unmethylated, gray zone, or methylated is presented in Table 2. There were 72 patients (10.5%) with values that fell into the gray zone that may be considered “slightly” methylated (Table 2). This is comparable to the proportion of gray zone patients observed in the study that established the cutoff and safety margin and reported 9.5% (82/863) in the training cohort and 8.1% (70/862) in the independent test cohort.^12^

**Table 2.** Classification of Patients by *MGMTp* Methylation Status Based on the Cutoff of 1.27 and the Safety Margin of −0.28

	Trial/treatment				
	Nordic/NOA8/RT (*N* = 196)	Nordic/NOA8/TMZ (*N* = 133)	CE.6/RT (*N* = 175)	CE.6/RT + TMZ (*N* = 183)	Total (*N* = 687)
	*N* (%)	*N* (%)	*N* (%)	*N* (%)	*N* (%)
*MGMTp status*					
Truly unmethylated	90 (45.9)	69 (51.9)	70 (40.0)	67 (36.6)	296 (43.1)
Gray zone	15 (7.7)	18 (13.5)	21 (12.0)	18 (9.8)	72 (10.5)
Methylated	91 (46.4)	46 (34.6)	84 (48.0)	98 (53.6)	319 (46.4)

#### Comparison of classification rules.—

The classification using the cutoff of 1.27, based on the corrected ratio, into methylated and unmethylated *MGMTp* samples was compared to the results using the original procedure and cutoff 1, also based on the corrected ratio. This confirmed good classification (Table 3). An almost perfect agreement was observed, with kappa values ≥ 0.9 in the pooled Nordic/NOA-08 and the CE.6 datasets (Table 3). In accordance, no significant survival differences were observed when comparing the data from the 2 classification procedures.

**Table 3.** Comparison of *MGMTp* Methylation Classification

	*MGMTp* classification based on cutoff of 1.27							
	Nordic/NOA8/RT		Nordic/NOA8/TMZ		CE.6/RT		CE6/RT + TMZ	
*MGMTp* status (original procedure and cutoff, 1)	Unmethylated (*N* = 105)	Methylated (*N* = 91)	Unmethylated (*N* = 87)	Methylated (*N* = 46)	Unmethylated (*N* = 91)	Methylated (*N* = 84)	Unmethylated (*N* = 85)	Methylated (*N* = 98)
Unmethylated	103 (98.1%)	5 (5.5%)	86 (98.9%)	0 (0%)	89 (97.8%)	7 (8.3%)	85 (100%)	8 (8.2%)
Methylated	2 (1.9%)	86 (94.5%)	1 (1.1%)	46 (100%)	2 (2.2%)	77 (91.7%)	0 (0%)	90 (91.8%)
Kappa (95% CI)	0.93 (0.86–0.97)		0.98 (0.92–1.00)		0.90 (0.81–0.95)		0.91 (0.83–0.96)	

#### Outcome of patients with truly unmethylated MGMTp and treatment.—

**Figure 2.**
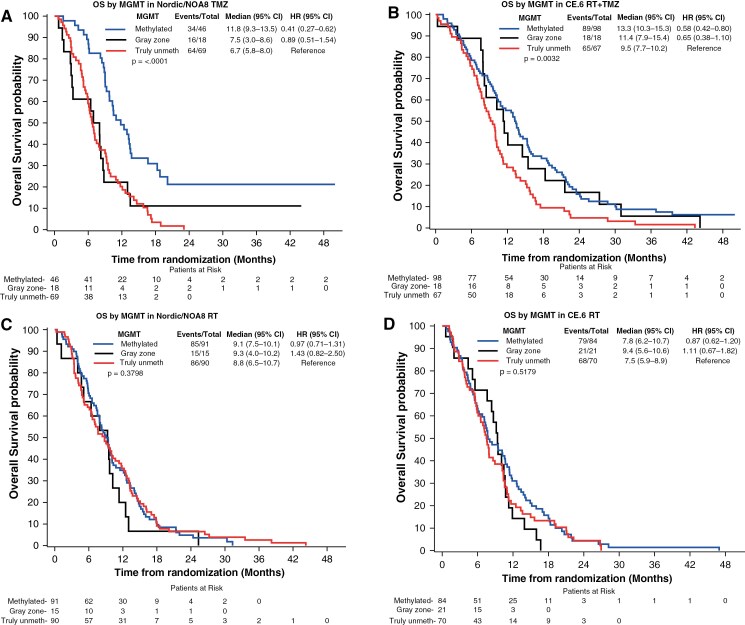
*MGMTp* methylation and OS based on cutoff and safety margin. The association of *MGMTp* methylation with OS in the trial populations, separated into *MGMTp* methylated (> 1.27), gray zone (−0.28 and 1.27), and truly *MGMTp* unmethylated patients (<−0.28) as indicated, stratified by treatment and illustrated in Kaplan–Meier plots. A significant difference was observed among the subgroups in the TMZ arms of the pooled Nordic/NOA8 trial (**A,***P*** **< .0001) and the CE.6 trial (**B,***P* = .0032), respectively (log-rank tests comparing all 3 curves). No differences were observed in the corresponding RT arms (**C, D).** Pairwise log-rank tests for results with significant OS differences are as follows: (**A**) Nordic/NOA8 TMZ: methylated versus gray zone, *P* = .005; methylated versus truly unmethylated, *P* < .001, gray zone versus truly unmethylated, *P* = .647. (**B**) CE.6 RT + TMZ: methylated versus gray zone, *P* = .618; methylated versus truly unmethylated *P* = .001; gray zone versus truly unmethylated, *P* = .096.

**Figure 3.**
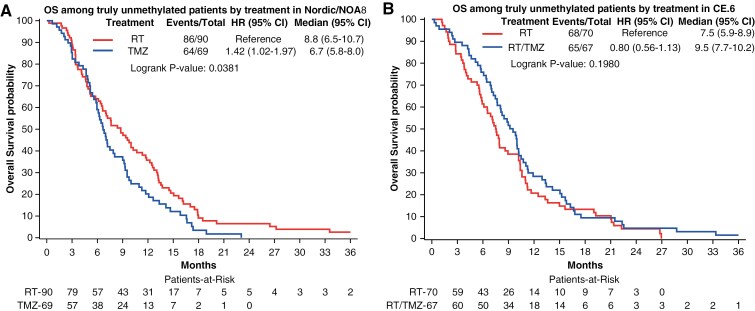
Patients with truly unmethylated *MGMTp* do not benefit from TMZ treatment. The OS of truly *MGMTp* unmethylated patients, split by treatment arm, is illustrated by Kaplan–Meier plots for the trial populations. (**A)** In the Nordic/NOA8 population, a significant difference was observed between TMZ treatment and RT (*P* = .0381). Shorter survival was observed for patients treated with TMZ. (**B)** The comparison of RT versus RT/TMZ→TMZ in the truly *MGMTp* unmethylated patients of the CE.6 trial showed no OS difference between treatments (*P* = .1980).

Survival analysis stratifying the patients into *MGMTp* methylated, truly unmethylated, and gray zone patients, is visualized in Figure 2 for both datasets (Nordic/NOA-08 and CE.6), separated by treatment. In the TMZ arms of the studies, the gray zone patients were situated between truly unmethylated and methylated survival curves (Figure 2A, B), although the small numbers preclude statistical conclusions. In the RT arms, no *MGMTp* status-dependent differences were observed (Figure 2C and D). A similar picture was observed when analyzing progression-free survival that was available for NOA-08 and CE.6, but not for the Nordic trial (Supplementary Figure S1). Subsequently, we evaluated OS in the truly *MGMTp* unmethylated patients (Figure 3A and B). The analysis of the pooled Nordic/NOA-08 cohort revealed a significantly better outcome in the patients treated with RT as compared to TMZ (P = 0.0381, unadjusted). Of note, when considering all unmethylated patients of the Nordic/NOA-08 cohort, this difference was not revealed (*P* = .17). Moreover, in the truly unmethylated patients of the CE.6 study, no difference in OS was observed between the RT and the RT/TMZ→TMZ arm (*P* = .19, unadjusted; Figure 3B), suggesting no benefit from the addition of TMZ to RT in this patient population. In the original analysis including all unmethylated patients, the comparison almost reached significance (*P* = .0551).

## Discussion

In this study, we reinvestigated the cutoff for *MGMTp* methylation with the aim of providing new evidence and tools for risk-adjusted treatment decisions in the management of elderly and frail GB patients. The previously established cutoff (1.27) and safety margin (−0.28) were revealed to be a good fit for the distribution of *MGMTp* methylation (ratio_c_) in this elderly patient population (median age of 72 years; Q1, Q3; 68.8, 75.0; Table 1). These cutoffs have been established previously in a pooled analysis of 4 clinical trials for adult GB patients treated with RT/TMZ→TMZ.^12^ The median age of this adult GB patient population was 57 years (Q1, Q3; 50, 63). This suggested that the *MGMTp* methylation cutoffs apply similarly to all adult GB patients. In this study, the cutoff of 1.27, which was developed in adult GB patients (median age of 57 years) was found to be applicable to this elderly GB patient population (median age = 72 years, ranging from 60 to 90 years). To this end, the cutoff of 1.27 applies to both adult and older GB patients, regardless of age, and may be generalized. Of note, the study populations discussed here were selected, fulfilling trial criteria, and may not reflect all elderly GB patients.

For elderly and frail patients, the question of how to best treat GB remains debated due to insufficient respective data according to the conclusions of recent reviews of the literature.^16–20^ The most recent guidelines from EANO and NCCN^21,22^ propose different treatment options for elderly (> 70 years) and/or frail GB patients (performance KPS < 60 or < 70), taking into account or not, the *MGMTp* methylation status of the GB. Based on the CE.6 study^13^ that showed almost significant benefit from the addition of TMZ to RT, many patients with tumors without *MGMT*p methylation receive combination therapy including TMZ with the hope of improving outcomes.

In the present study, we provide evidence that patients can be identified, who do not benefit from treatment with TMZ. Re-analyzing the 3 phase III trials including specifically elderly GB patients, we demonstrated that the application of a previously established restrictive cutoff allowed the identification of truly *MGMTp* unmethylated patients, who do not benefit from TMZ treatment. This implies that TMZ can be safely omitted in these patients, avoiding undue TMZ-related toxicity^4,5,13,23^ and costs, without compromising the outcome. In contrast, for patients whose *MGMTp ratio*_*c*_ falls into the gray zone (“low” *MGMTp* methylation), TMZ should be added to RT in order not to withhold a potentially effective treatment. Accordingly, only patients with truly unmethylated *MGMTp* should be randomized into trials omitting TMZ in the test arm and excluding patients in the gray zone. When single treatment modalities are considered, TMZ treatment alone may be an option for patients with clearly *MGMTp* methylated GB, otherwise, patients should receive RT.

Along the same lines, it will be of interest to re-analyze recent clinical trials that selected GB patients with unmethylated *MGMTp* to omit TMZ in the test arm. We hypothesize that similar to the CE.6 trial, patients from the gray zone may give the false signal of a benefit from the addition of TMZ to RT. The trial testing Nivolumab in combination with RT against standard-of-care treatment containing TMZ, has used the same qMSP assay, using the technical cutoff at the nadir. Applying the restrictive cutoff with the safety margin would allow removing the patients in the gray zone from the analysis, who in the control arm may have benefitted from TMZ treatment, leading to an apparent detrimental result in the test arm.^9^

We are aware that the qMSP assay discussed in this study has been used centrally for most large clinical trials for GB using a commercial service^24–27^ (eg, Labcorp, https://www.labcorp.com/), and may therefore not be available at local pathologies. However, any assay has uncertainty of classification close to the assay-specific cutoff for biological and/or technical reasons. This “gray zone” comprises patients with low methylation or a methylation pattern that is not properly detected by a given assay. Intermediate outcomes have been reported for patients with intermediate methylation.^28,29^ There are different technologies available for *MGMTp* methylation testing besides qMSP, such as methylation-specific pyrosequencing, the EPIC DNA methylation array, and others.^30,31^ However, no consensus for assays and respective cutoffs has been reached, see recent reviews detailing pro and contra of specific assays and respective recommendations for *MGMT*p methylation testing.^32,33^ For some assays, a respective gray zone has been defined, validated, and implemented for best prediction of outcome in GB patients.^12,29,30^ The definition and validation of assay-specific gray zones are warranted, in order to improve the selection of truly *MGMTp* unmethylated patient, when considering omitting TMZ, e.g. in platform trials.^7,8^ On the other hand, criteria for truly *MGMTp* methylated GB need to be established, eg, when adding another alkylating agent such as CCNU, to promise benefit in order to justify the added toxicity.^34^ In the qMSP assay discussed in this study, the cutoff for truly *MGMTp* methylated overlapped with the cutoff at the nadir of 1.27, as determined by OS supervised analysis in a large cohort of RT/TMZ→TMZ treated GB patients.^12^

In conclusion, the cutoff to be applied depends on the clinical question. Respective validated, assay-specific cutoffs are required for stratified therapy in clinical trials and evidence-based management of all GB patients.^33^

Author collaborations

Nordic Clinical Brain Tumour Study Group (NCBTSG) (R.H., A.M.), Canadian Cancer Trials Group (CCTG) (J.R.P., N.J.L.), Neuro-oncology Working Group (NOA) of the German Cancer Society (W.W., M.W.), European Organization for Research and Treatment of Cancer - Brain Tumour Group (EORTC-BTG) (M.E.H., F.B.O., W.W., R.H.,T.G., A.M.,

## Supplementary material

Supplementary material is available online at *Neuro-Oncology* (https://academic.oup.com/neuro-oncology).

noae108_suppl_Supplementary_Figures_S1

## Data Availability

The data can be requested at Data And Sample Sharing—EORTC (https://www.eortc.org/data-sharing/).
