# Supplementary material for: No benefit from TMZ treatment in glioblastoma with truly unmethylated MGMT promoter: Reanalysis of the CE.6 and the pooled Nordic/NOA-08 trials in elderly glioblastoma patients
Source: Neuro Oncol. 2024 Jul 3;26(10):1867–75. doi: 10.1093/neuonc/noae108 (PMC11449086; doi:10.1093/neuonc/noae108)
Supplement: noae108_suppl_Supplementary_Figures_S1 [file noae108_suppl_supplementary_figures_s1.docx]

**Supplementary Figure S1**. *MGMTp* methylation and PFS based on cutoff and safety margin.

The association of *MGMTp* methylation with progression free survival (PFS) in the trial populations, separated into *MGMTp* methylated (>1.27, blue), gray zone (-0.28 and 1.27, black), and truly *MGMTp* unmethylated patients (< -0.28, red), stratified by treatment and illustrated in Kaplan–Meier plots. A significant difference was observed among the subgroups in the TMZ arms of the NOA8 trial (**A,** p < 0.0001) and the CE.6 trial (**B,** p=0.0014), respectively (log rank tests comparing all three curves). No differences were observed in the corresponding RT arms (**C, D).** Of note, PFS was defined differently in the NOA-08 and the CE.6 trials: In NOA-08, PFS was measured from the date of randomization until disease progression or death (if no progression was reported) or until the date of last visit. In CE6, progression-free survival was measured from the date of randomization until disease progression or death (if no progression was reported) or until the last evaluation date. No PFS data was collected in the Nordic trial. Pairwise log-rank tests for results with significant PFS differences are as follows: (**A**) NOA8 TMZ: methylated vs gray zone, p = 0.138; methylated vs truly unmethylated, p < 0.001, gray zone vs truly unmethylated, p = 0.027. (**B**) CE.6 RT+TMZ: methylated vs gray zone, p = 0.090; methylated vs truly unmethylated p < 0.001; gray zone vs truly unmethylated, p = 0.387.
